# Minocycline treatment reduces the activation of mononuclear phagocytes and improves retinal function in a mouse model of Leber congenital amaurosis

**DOI:** 10.1007/s00417-025-06768-y

**Published:** 2025-06-18

**Authors:** Ettel Bubis, Ifat Sher, Hadas Ketter-Katz, Estela Derzane, Florian Sennlaub, Ygal Rotenstreich

**Affiliations:** 1 Goldschleger Eye Institute, Sheba Medical Center, Tel-Hashomer, 5262100 Israel; 2 Ophthalmology Department, Faculty of Medical and Health Sciences, Tel-Aviv University, Tel Aviv, 6997801 Israel; 3 The TELEM Rubin Excellence in Biomedical Research Program, Sheba Medical Center, Tel Hashomer, 5262100 Israel; 4 Institut de la Vision, 17 rue Moreau, Paris, 75012 France; 5 Sorbonne Universités, UPMC University Paris 06, INSERM, CNRS, Paris, 75012 France; 6 Sagol School of Neuroscience, Tel Aviv University, Tel Aviv, 6997801 Israel; 7 Academic Center for Continuing Medical Education, Faculty of Medicine and Health Sciences, Tel Aviv University, Tel Aviv, Israel

**Keywords:** Mononuclear phagocyte, Retinitis pigmentosa, LCA, RPE65, Minocycline

## Abstract

**Purpose:**

Leber congenital amaurosis (LCA) is a severe hereditary retinal degeneration characterized by early-onset vision loss. Here, we aimed to characterize the association between retinal mononuclear phagocyte (MP) activation and retinal degeneration in the *RPE65/rd12* mouse model of LCA.

**Methods:**

Thirty-nine *RPE65/rd12* and ten *C57BL/6J* wild-type mice were used. *RPE65/rd12* mice were treated with minocycline by daily intraperitoneal injection (5 mg/kg) for eight weeks starting at age postnatal day 28 (P28). MP cell density in the subretina was determined by choroid-retinal pigment epithelium (RPE) flat mount analysis, and retinal function was determined by electroretinogram (ERG).

**Results:**

In wild-type *C57BL/6J* mice, MPs were exclusively located in the inner retinal layers at ages P28-P84. By contrast, in the *RPE65/rd12* mice, MPs migrated into the subretina as early as P56 in a central-to-peripheral gradient. By P84, the density of MPs in the subretina increased by nearly 3-fold, reaching 61.3 ± 6.2 cell/mm^2^ and 33.1 ± 8 cell/mm^2^ in the central and peripheral retina, respectively. Minocycline treatment significantly reduced MP density in the peripheral subretina (16.2 ± 1.8 MP cell/mm^2^) compared with mice treated with PBS (27.2 ± 2.4 MP cell/mm^2^, respectively, *p* = 0.006). Maximal electroretinogram b-wave responses were significantly higher in minocycline- vs. PBS-treated mice under light-adapted conditions following eight weeks of treatment (mean ± SE: 199µv ± 28µv vs. 129.8µv ± 9.8µv, *p* = 0.016).

**Conclusions:**

Our data indicates that MP migration into the subretina is associated with retinal degeneration in* RPE65/rd12* mice. Inhibiting MP migration into the subretina was associated with improved retinal function. These findings may guide the development of therapies targeting MP activation for neuroprotection in LCA and potentially other retinoid cycle-related retinal degeneration blinding diseases.

**Supplementary Information:**

The online version contains supplementary material available at 10.1007/s00417-025-06768-y.

## Abstract (highlights)

**Key messages:**

*****What is known***:**

- Leber congenital amaurosis (LCA) is a severe hereditary retinal degeneration characterized by early-onset vision loss.

*****What is new***:**

- Retinal mononuclear phagocyte (MP) migration into the subretina was associated with reduced retinal function in the *RPE65/rd12* mouse model of LCA.
- MPs migrated into the subretina of the mice in a central-to-peripheral gradient.
- Minocycline treatment inhibited the migration of MPs into the subretina and improved retinal function.

**Supplementary Information:**

The online version contains supplementary material available at 10.1007/s00417-025-06768-y.

## Introduction

Retinitis Pigmentosa (RP) is the most common cause of hereditary untreatable neuroretinal degeneration, affecting 1:3500-1:4000 births. It is a highly genetically heterogeneous disease in which rod photoreceptors’ initial loss is followed by cone degeneration and the RPE [1]. Leber congenital amaurosis (LCA) is a severe form of RP characterized by early-onset degenerative retinal dystrophy [2, 3].

Mononuclear phagocyte (MP) activation is well documented in neurodegenerative diseases of the central nervous system, including Alzheimer’s disease, Parkinson’s disease, amyotrophic lateral sclerosis, Huntington’s disease, and acute spinal cord trauma [reviewed in [4–6]. Studies in animal models suggest that activation of MPs plays a role in retinal neurodegeneration in age-related macular degeneration (AMD [7, 8]), RP [9–13], and LCA [14, 15]. Activated MPs were identified in the outer nuclear layer (ONL) of AMD and RP patients, where they were suggested to contribute to the degeneration of photoreceptors [7, 11, 16]. Hyperreflective dots, that were attributed to activated microglia were identified using Spectral Domain-Optical Coherence Tomography (SD-OCT) across retinal layers, with higher abundance in the outer retinal layers and in proximity to fluid accumulation areas in wet AMD patients [17]. Studies on retinal detachment and light-induced retinal degeneration models indicated that MPs clear cell debris, secrete neurotrophic factors, and promote photoreceptor survival [18–21]. Endogenous bone marrow (BM)-derived MPs were shown to play a protective role in the *rd1* and *rd10* mouse models, decelerating retinal degeneration and promoting cone cell survival [22]. In addition, in the slow retinal degeneration *rd10* mouse model, MP activation was essential for the anti-apoptotic effect of IGF-I [23].

However, in vitro and in vivo studies demonstrated that the MPs have a dual role in the progression of photoreceptor degeneration. Several studies reported that MPs release neurotoxins and cytokines (e.g., NO, ROS, TNF, Il-6, IL-1β) that induce apoptosis of photoreceptors [24, 25]. Activated MPs clear the apoptotic photoreceptors and their healthy neighboring cells, exacerbating photoreceptor cell loss [26]. In several retinal dystrophy rodent models, including mice with mutations in the *PDE6b* gene, rats with *rhodopsin* gene mutations, and the *Cngb1−/−* mice, MP activation was shown to precede photoreceptor cell loss and treatments that reduced the activation of MPs ameliorated photoreceptor degeneration, suggesting that MPs may initiate and or accelerate retinal degeneration [9, 26–28]. Those studies suggest that therapies modulating MP cytotoxic effects at the correct timing, duration, and dosage may represent a new therapeutic strategy for treating retinal degeneration.

Minocycline is a semisynthetic tetracycline derivative widely used to treat gram-positive and gram-negative infections [29]. Due to its high lipid solubility, minocycline can cross the blood-brain barrier [30, 31]. It was also shown to selectively inhibit MP activation, reduce MP migration into the subretina, lower the secretion of inflammatory cytokines such as TNF-α and IL-1β and deaccelerate photoreceptor loss and RPE cell death in several rodent models of retinal degeneration [32–38].

Retinal pigment epithelium 65 (RPE65( is one of the enzymes of the retinoid cycle that is essential for the conversion of all-trans-retinol to the vision chromophore 11-cis retinal, required for the proper function of both rods and cones. Mutations in the *RPE65* gene cause RP and LCA type 2 associated with severe vision loss at a young age [2]. This gene gained significant public attention as the FDA approved a gene therapy (LUXTURNA) for patients with *RPE65* loss of function [39–41].

The *RPE65/rd12* mouse model carries a nonsense mutation in exon 3 of the *RPE65* gene and is a well-characterized model for *RPE65* deficiency [42]. Loss of RPE65 expression leads to dysfunction of the retinoid cycle and reduced levels of 11-cis retinal in the photoreceptors, resulting in slow retinal degeneration [42]. Cone degeneration begins at an early age in these mice in dorsal-to-ventral gradient, with loss of the majority of the cones in the central, ventral, and nasal quadrants of the retina by 35 days postnatal (P35) [43, 44]. Even though the ONL thickness appears nearly normal, electroretinogram (ERG) testing demonstrated substantially reduced responses to light stimuli compared to WT mice, as early as P21 [42, 45, 46].

Sasahara et al. have demonstrated that BM-derived cells were recruited to the degenerating retina in *RPE65/rd12* mice, where they differentiated into MPs and subsequently localized to the degenerating vessels and neurons [22]. However, to our knowledge, the course of MP activation in this mouse model was not determined. Moreover, the therapeutic effects of MP inhibition in this model remain unknown. We hypothesized that MP activation accelerates retinal degeneration in the *RPE65/rd12* mouse model and that inhibiting MP activation may provide neuroprotective effects and promote photoreceptor survival and function.

## Methods

### Animals

*RPE65/rd12* mice (*n* = 63) were born and bred in the Sheba Medical Center animal facility under dim cyclic light (12 h at < 5 lx, 12 h in the dark). *C57BL/6J* mice (*n* = 10) were purchased from Envigo (Rehovot, Israel). Ten *RPE65/rd12* mice were used to determine the MP density in the sub retina on postnatal days 28 (P28), 56 (P56), and 84 (P84). Twenty-five *RPE65/rd12* mice received daily intraperitoneal injections (100 µL) of minocycline (5 mg/Kg) and 23 *RPE65/rd12* mice received daily intraperitoneal injections (100 µL) of PBS as control. All animal procedures and experiments were approved by the Sheba Medical Center Institutional Animal Care Committee and conformed to recommendations of the Association for Research in Vision and Ophthalmology (ARVO) Statement for the Use of Animals in Ophthalmic and Vision Research and according to the Animal Research: Reporting of In Vivo Experiments (ARRIVE).

### Immunofluorescence analysis

For immunofluorescence analysis, eyes were fixed in 4% paraformaldehyde (PFA) and embedded in sucrose for cryopreservation. Eight-micrometer sections along the vertical meridian of the eye through the optic nerve were cut using a cryostat. Sections were pretreated with a blocking solution (1% BSA, 5% Normal Goat Serum, and 0.5% Triton- in PBS) for three hours at room temperature, followed by incubation with an antibody directed against the Iba-1 (Rabbit anti Iba1, 019–19741, Wako chemicals, USA, 1:500 in PBS containing 0.3% triton-x and 1% BSA) for 16 h at 4°C. Iba-1 is a well-established marker for MPs [47, 48]. Following excessive washes in PBS, sections were incubated with Alexa Fluor^®^ 488-AffiniPure Donkey Anti-Rabbit IgG antibody (Jackson Immuno Research, USA) in a 1% BSA solution for 1 h at room temperature. The sections were counterstained with 4’,6-diamidino-2-phenylindole (DAPI).

### RPE flat mount preparation and analysis

Eyes were lightly fixed in 4% PFA for 40 min. The cornea, iris, and lens were removed, and the remaining cup was dissected into four petals [49]. The neuro-retina was peeled off and the RPE flat mount was incubated with an Iba-1 antibody (Rabbit anti-Iba1, 019–19741, Wako chemicals, USA, 1:200 in PBS and 0.1% Triton-X) for 16 h at 4^o^C, followed by incubation with secondary Alexa Fluor^®^ 488-AffiniPure Donkey Anti-Mouse IgG antibody (Jackson Immuno Research; USA, 1:400 in PBS with 0.2% DAPI) for 2 h at room temperature.

### Microscopy analysis

**Fig. 1:**
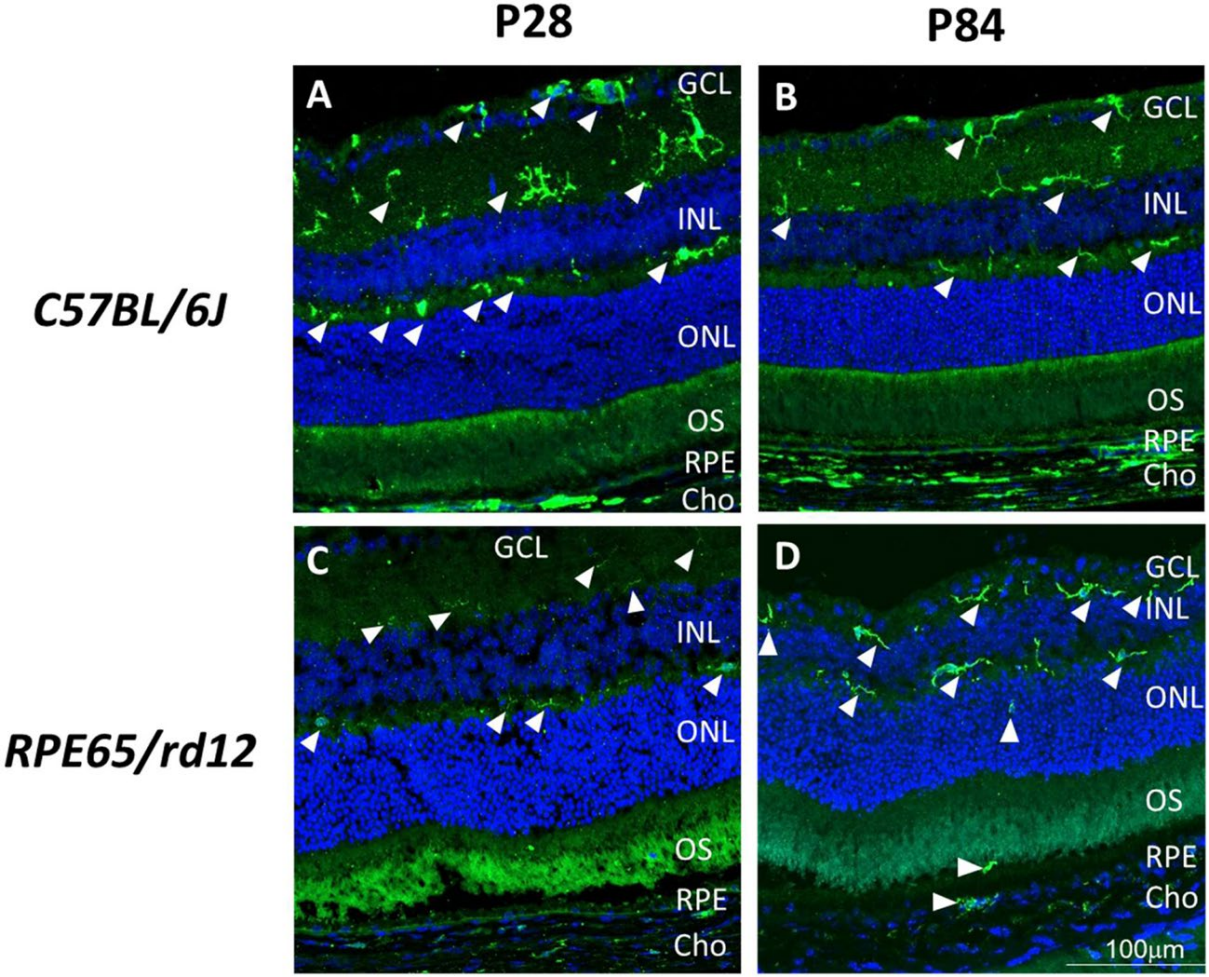
MP activation in *RPE65/rd12* mice. Retinal sections of *RPE65/rd12* (**A**, **B**) and WT *C57BL/6J* (**C**, **D**) mice at post natal day 28 (P28, **A**, **C**) and P84 (**B**, **D**) were stained with an antibody directed against Iba-1 (Rabbit anti-Iba1, 019–19741, Wako chemicals, USA, green) and counter-stained with DAPI (blue). White arrowheads highlight MPs. At least three mice were used for each time point. GCL- Ganglion Cell Layer; INL- Inner Nuclear Layer; ONL- Outer Nuclear Layer; OS- Outer Segments; RPE- Retinal Pigmented Epithelium; Cho- Choroid. Scale – 100 μm

Images of retinal sections (Fig. 1) were obtained with a confocal microscope (Zeiss LSM700) using a 20X objective, digital zoom 2x.

**Fig. 2:**
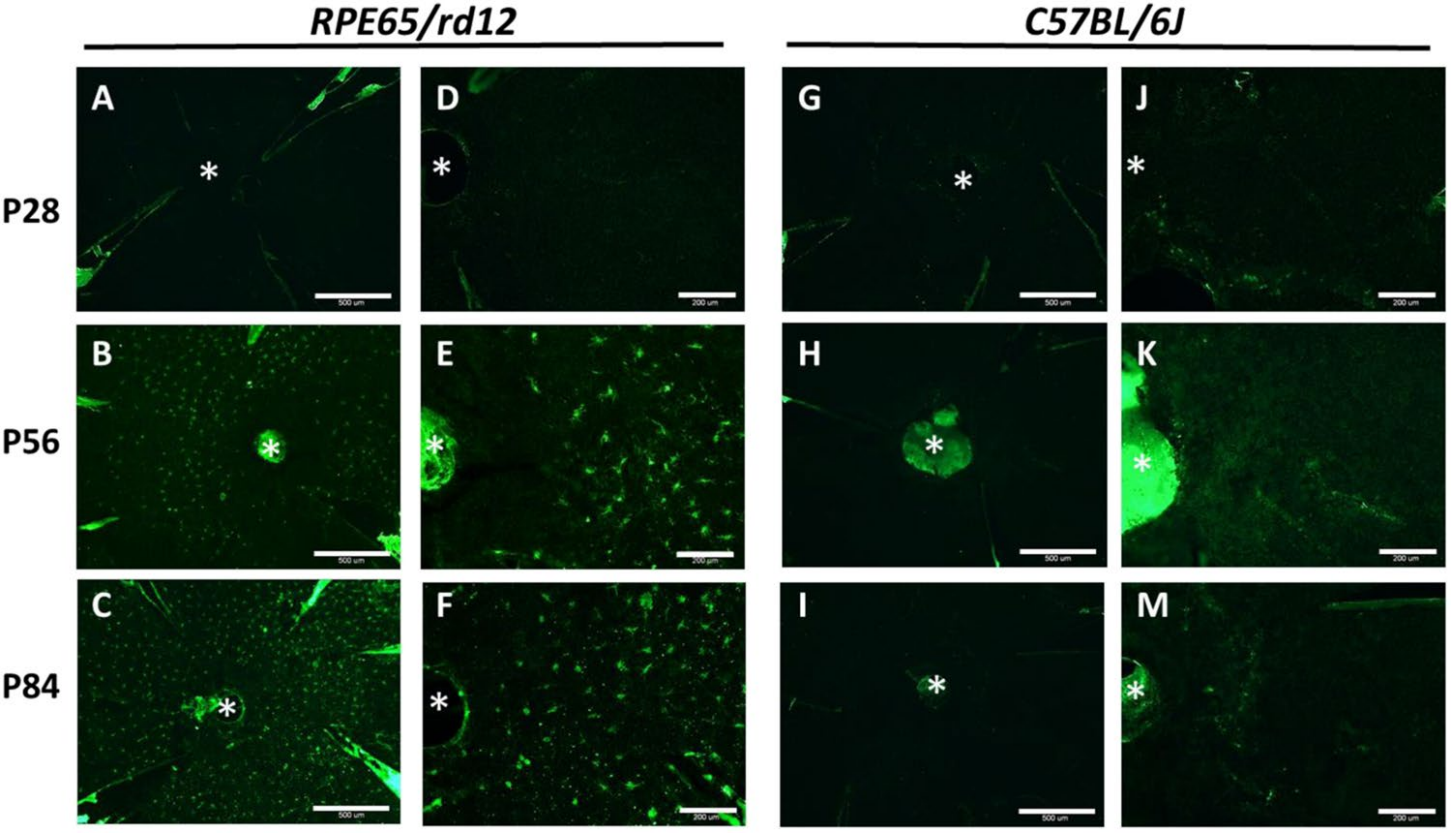
MP infiltration into the subretina. Retinal pigment epithelium (RPE) flat mounts of *RPE65/rd12* (**A**-**F**) and *C57BL/6J* mice (**G**-**L**) were stained with an antibody directed against Iba-1 (Rabbit anti-Iba1, 019–19741, Wako chemicals, USA, Green). In panels **A**-**C **and **G**-**I**, the scale bar = 500 μm. Panels **D**-**F **and **J**-**L **show a higher magnification of the same RPE flat mounts presented at panels **A**-**C **and **G**-**I**, respectively (scale bar = 200 μm). The optic nerve head is marked with an asterisk. At least three mice were used for each time point

**Fig. 3:**
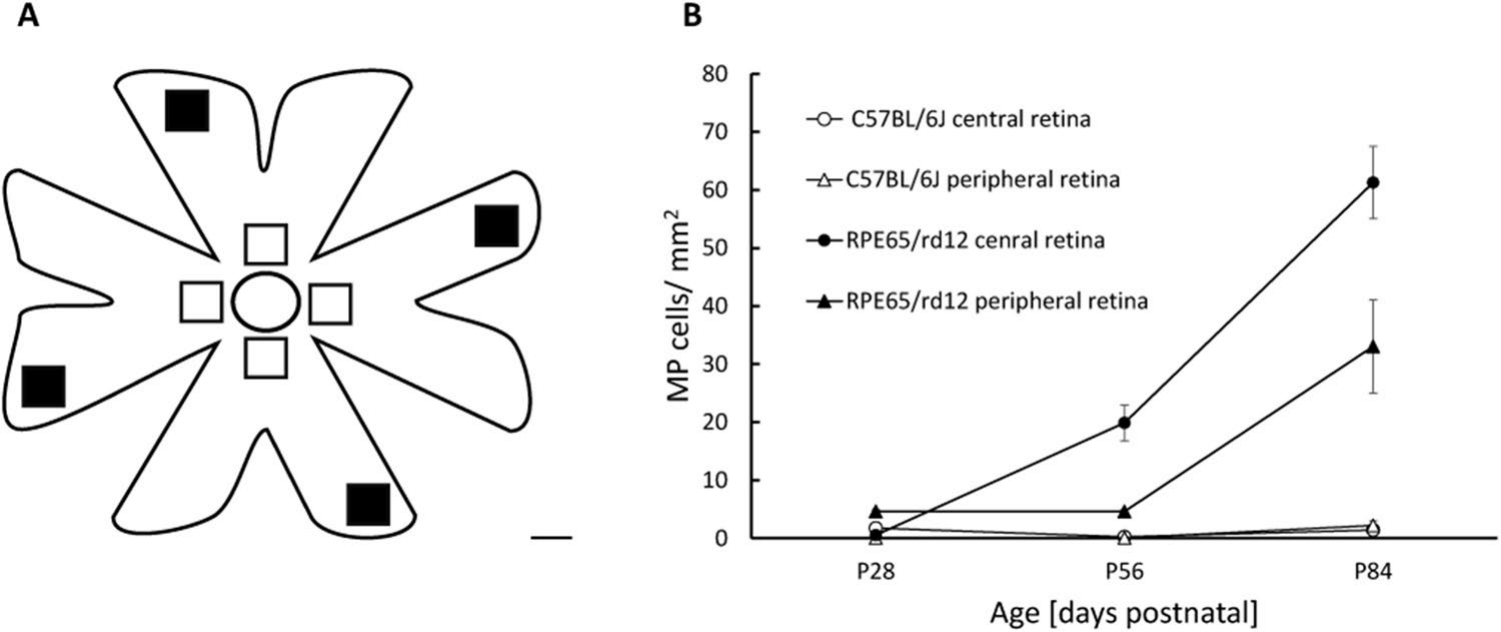
Quantification of MP cell density in the subretina. **A**- Schematic demonstration of the RPE flat-mount. The one mm^2^ images taken for quantifying MPs in the central retina are marked in white squares, and the one mm^2^ images taken for quantifying MPs in the peripheral retina are marked in black squares. Scale bar = 1 mm. The optic nerve head is shown as a circle. **B-** The density of Iba-1 positive cells in the RPE flat mount was quantified in WT and *RPE65/rd12* mice at indicated ages using *Image J*. Three RPE flat mounts were evaluated for each group in each time point. Data are presented as mean ± standard error (SE)

All flat-mount samples (Figs. 2 and 3) were visualized using a fluorescent microscope (Olympus BX51), with 40X or 100X objectives, and recorded using an Olympus DP71 camera. To assess the number of MPs that have migrated into the subretinal space, we used images centered on the visual disc. Iba-1 positive cells bearing the distinct MP morphology were identified [50] and manually counted by two masked observers (EB and HKK).

*Image J* (National Institutes of Health, Bethesda, MD) was used for all image processing. All analyses were masked.

### Electroretinogram (ERG)

Full-field ERG measures the mass electrical response of the retina to photic stimulation. The amplitudes of the b-wave, which is generated by the bipolar cells of the inner retina and is the most commonly used parameter in clinical studies and animal research, and the a-wave, which is generated by the cones and rods in the outer photoreceptor layer, were measured [51–53]. For dark-adapted ERG, mice were dark-adapted for 12 h before testing. Animals were anesthetized with intraperitoneal injections of 75 mg/Kg Ketamine and 10 mg/kg Xylazine, pupils were dilated with topical 1% tropicamide (Mydramid, Fischer Pharmaceutical, Israel), and the corneas were kept moist with 2.5% hydroxypropyl methylcellulose (Celluspan, Fischer Pharmaceutical, Israel). ERG was recorded from both eyes simultaneously using silver wire loops on the corneas in response to light stimuli at five increasing intensities (0.25, 4.4, 23.5,141 and 655 cd-s/m^2^) as we previously described [53–55]. All analyses were masked.

### Statistical analysis

A two-tailed t-test was used to evaluate MP subretinal density without assuming equal variance. For assessment of the effect of minocycline treatment on ERG response, the treatment effect was assessed at P84 using a generalized linear model (GLM) repeated measures (full factorial) models that were built as follows: the “between-factor” (between-group) was between-treatments (minocycline or PBS) and the “within-factor” was the five light stimulus intensities. The Mann-Whitney test was performed to evaluate the difference in weight gain between groups. All analyses were performed with IBM SPSS Statistics for Windows, version 25.0. Armonk, NY: IBM Corp. *P*-value ≤ 0.05 was considered statistically significant.

## Results

### MP activation and migration in *RPE65/rd12* mouse retina

First, we compared the localization of MPs in the retina of *RPE65/rd12* and control (*C57BL/6J*) mice on age postnatal day 28 (P28), by staining retinal sections with an antibody directed against the MP marker Iba-1. At P28, MPs were confined to the inner retina, with Iba-1 positive cells observed in the ganglion cell layer (GCL) and the inner plexiform layer in both mouse strains (Fig. 1A & C). At P84, Iba-1 positive cells were also identified in the photoreceptor nuclear layer (ONL) outer segment (OS) and choroid layers (Fig. 1D) in the *RPE65/rd12*. By contrast, in the WT mice, MPs were confined to the inner retina at P84 (Fig. 1B).

Next, we evaluated MP cell density in the subretina in RPE flat mounts. No MPs were detected in the subretina of control or *RPE65/rd12* mice at P28 (Fig. 2A, D, G, J). At P56 and P84, numerous Iba-1 positive cells were identified in the subretina of *RP65/rd12* mice (Fig. 2B, C, E, F) but not in the subretina of control WT mice (Fig. 2H, K, I, M).

Figure 3 demonstrates the quantification of MP cell density in the central and peripheral subretina. The number of MPs in the peripheral retina was determined by averaging the number of MPs in 1 mm^2^ images taken within 2 mm of the RPE flat mount edges in the four petals (black squares, Fig. 3A). The number of MPs in the central retina was determined by averaging the MP cell density from images taken within 2 mm of the optic nerve head in each petal (open squares, Fig. 3A). At P28, hardly any MPs were identified in the central (1.7 ± 0.66 cell/mm^2^ vs. 0.55 ± 0.2 cell/mm^2^, respectively, mean ± SE, *p* = 0.26) or peripheral (0.88 ± 0.21 cell/mm^2^ vs. 1.52 ± 0.57 cell/mm^2^, respectively, *p* = 0.19) retina of WT and *RPE65/rd12* mice, respectively.

A significantly higher MP cell density was observed in the *RPE65/rd12* compared with WT mice at P56 (central retina: 19.9 ± 3.1 cell/mm^2^ vs. 0.2 ± 0.1 cell/mm^2^, *p* = 0.035; peripheral retina: 4.6 ± 0.3 cell/mm^2^ vs. 0.1 ± 0.1 cell/mm^2^, *p* = 0.004, respectively) and at P84 (central retina: 61.3 ± 6.2 cell/mm2 vs. 1.4 ± 0.3 cell/mm^2^, *p* = 0.015; peripheral retina: 33.1 ± 8 cell/mm^2^ vs. 2.2 ± 0.8 cell/mm^2^, *p* = 0.013, respectively).

A significantly higher MP cell density was observed in the central vs. peripheral retina in the *RPE65/rd12* mice at P56 (19.9 ± 3.1 cell/mm^2^ vs. 4.6 ± 0.3 cell/mm^2^, respectively, *p* = 0.026) and P84 (61.3 ± 6.2 cell/mm^2^ vs. 33.1 ± 8 cell/mm^2^, respectively, *p* = 0.009).

### Inhibition of MP activation is associated with enhanced retinal function in *RPE65/rd12* mice

**Fig. 4:**
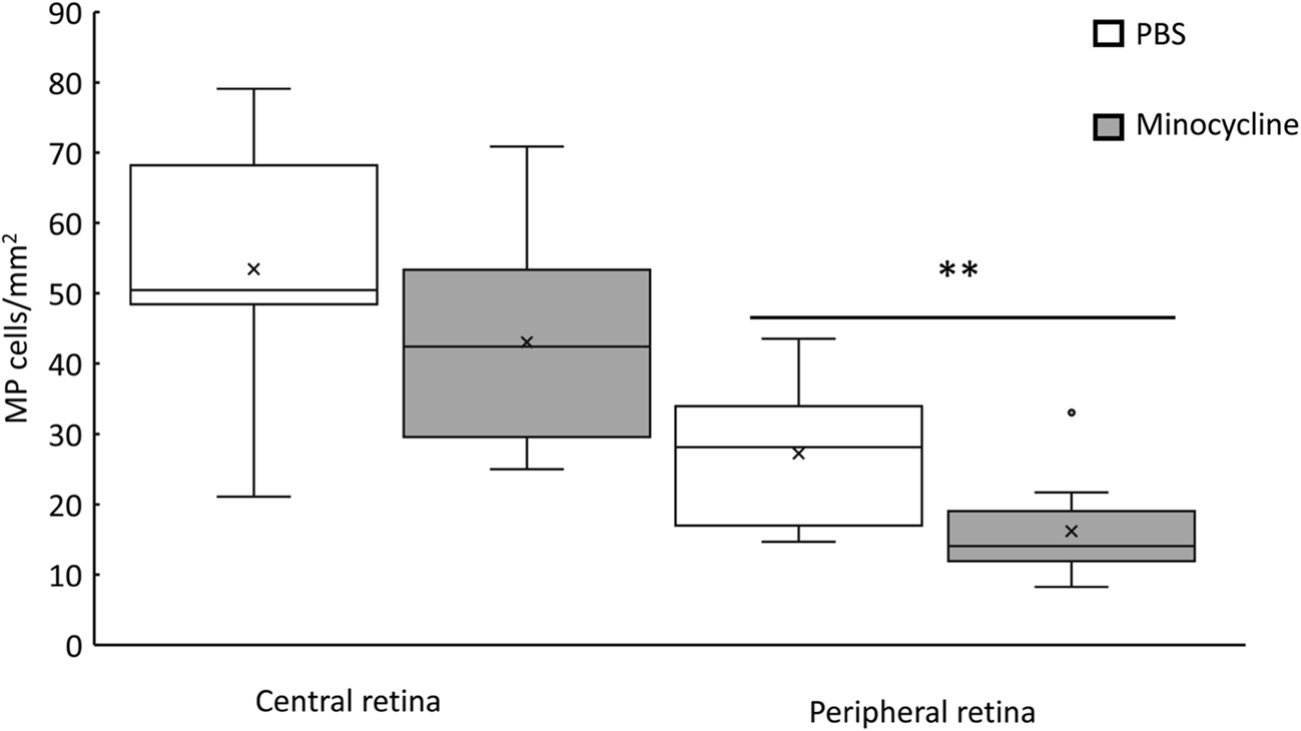
Minocycline treatment inhibits MP migration into the subretina in *RPE65/rd12* mice. MP cell density in the RPE flat-mount was determined as indicated in Fig. 3 in *RPE65/rd12* mice treated for eight weeks with PBS (*n* = 11) or minocycline (*n* = 10). The boxes represent the interquartile range (IQR, the difference between the first and the third quartile) of MP density in each group. The horizontal line represents the median. An X represents the mean value, and the whiskers extend to the maximal and minimal values. ** *P* < 0.01

To assess the association between MP activation and retinal degeneration in *RPE65/rd12* mice, the mice were treated with daily intraperitoneal injections of minocycline (5 mg/Kg) or the same volume of PBS. Following eight weeks of treatment, the retinas were harvested for RPE flat mount analysis and were stained with Iba1 antibody. As shown in Fig. 4, MP density in the peripheral subretina was lower by 41% in mice treated with minocycline for eight weeks compared with mice treated with PBS (mean ± SE: 27.26 ± 2.4 vs. 16.19 ± 1.84, respectively, *p* = 0.006). Minocycline treatment had a milder effect on MP infiltration into the central subretina. Thus, mice treated with minocycline presented with 20% lower MP subretinal density in the central retina than PBS-treated mice (Mean ± SE: 53.4 ± 4.04 vs. 43 ± 4, respectively, *p* = 0.14).

**Fig. 5:**
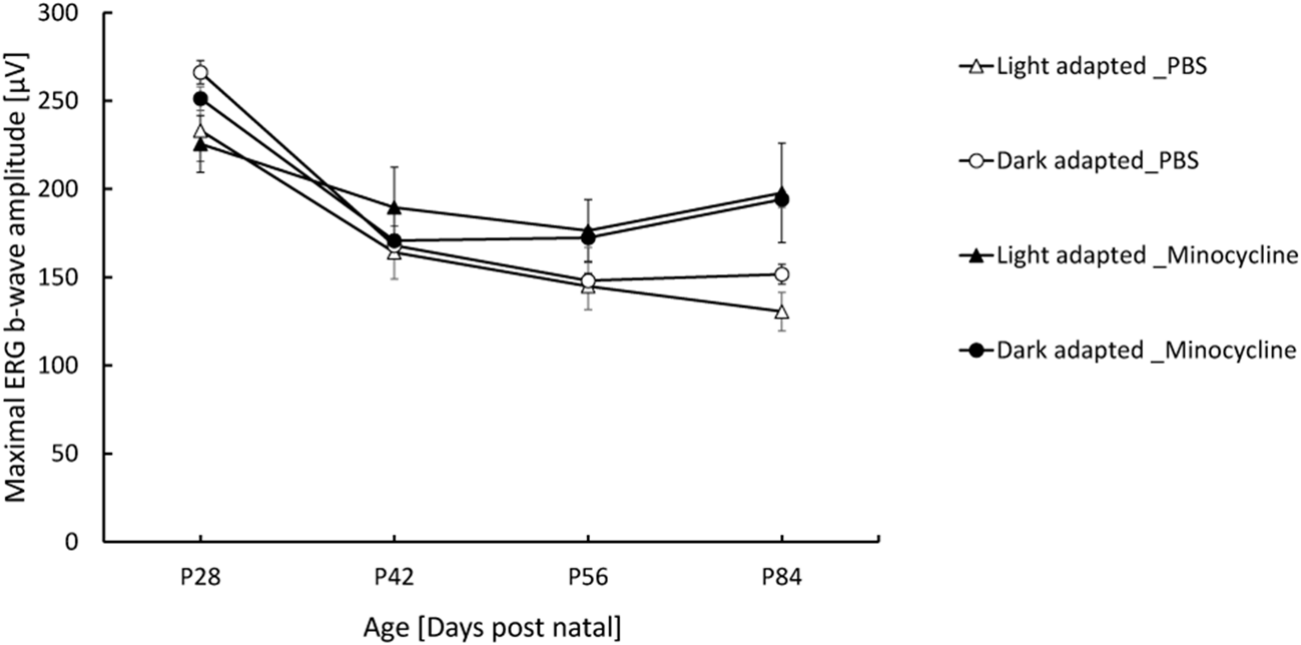
Minocycline treatment enhances retinal function in *RPE65/rd12* mice. Maximal ERG b-wave was recorded in minocycline- and PBS-treated mice under dark- and light-adaptation conditions. Data are presented as mean ± standard error (SE). The number of mice in each group: minocycline- *n* = 19 at postnatal day 28 (P28) and P42, *n* = 18 at P56, and *n* = 11 at P84. PBS- *n* = 17 at P28-P56 and *n* = 16 in P84

The *RPE65/rd12* mice underwent ERG testing before treatment commencement (at P28) and at ages P42, P56, and P84 (2, 4, and 8 weeks of treatment, respectively). Following eight weeks of treatment, the mean maximal b-wave ERG recorded was significantly higher in minocycline vs. PBS-treated mice under light adaptation (197.9µV ± 28.2µV vs. 130.5µV ± 10.9µV, respectively, *p* = 0.023, Fig. 5). A higher mean maximal b-wave ERG was recorded in minocycline vs. PBS-treated mice under dark adaptation. Still, this difference did not reach statistical significance (194.2µV ± 22.9µV vs. 151.7µV ± 14µV, *p* = 0.33). Representative ERG waveforms are shown in Supplementary Figs. 1 and 2.

**Fig. 6:**
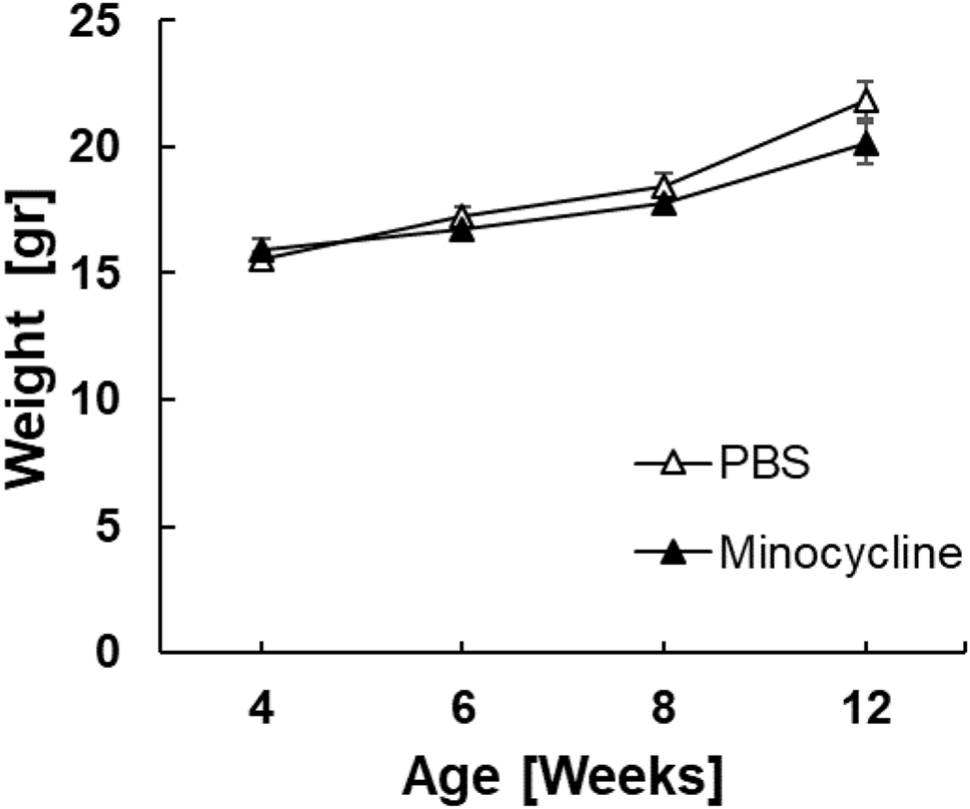
*RPE65/rd12* mouse weight. Mice were weighed once weekly. Data are presented as mean ± standard error (SE). The number of mice in each group: minocycline: *n* = 25 at postnatal day 28 (P28) and P42, *n* = 24 at P56 and *n* = 15 at P84. PBS: *n* = 23 at P28-P56, and *n* = 17 at P84

To evaluate the safety of systemic minocycline treatment, the weight of the mice was monitored by weekly weighing throughout the experiment. No significant differences in the mouse weight between the minocycline and PBS groups were identified (Fig. 6, all *p* > 0.071).

## Discussion

This study demonstrates that MP activation and migration into the subretina is associated with retinal degeneration in mice lacking RPE65 protein expression. Activated MPs were identified in the subretinal space in the *RPE65/rd12* mice at 56 days postnatal. Minocycline treatment reduced MP activation and migration into the subretina and enhanced retinal function. These findings suggest that MP activation may play a role in retinal degeneration in this mouse model. Treatments targeting MP activation may potentially provide a neuroprotective effect in RP associated with RPE65 mutations.

Retinal degeneration progression is slow in the *RPE65/rd12* mice. Our ERG recordings demonstrated a gradual loss of photoreceptor function, but the ERG responses were still recordable by P84. These findings are in accordance with our previous studies and studies published by other groups, demonstrating that by the age of two years, the ONL contains several nuclear layers, and ERG is still recordable, though substantially reduced by the age of 14 months in these mice [42, 44, 45, 53].

In acute retinal degeneration mouse models, such as models of light damage, the rods rapidly degenerate, and the number of MPs in the sub-retina is in the range of several hundred Iba1^+^cells/mm^2 [33]. By contrast, in the chronic slow-progressing retinal degeneration *PRE65/rd12* mouse model used in our study, the number of Iba1^+^ cells/mm^2 was substantially lower, reaching ~ 70 Iba1^+^ cells/mm^2 in the peripheral subretina (Fig. 3). Other groups obtained similar results by using other slow-degenerating mouse models. For example, ~ 80 Iba1^+^cells/mm^2 retina were observed in the *Cngb1−/−* mouse model at age P28, a four-fold higher number of Iba1 + cells compared to WT mice (~ 20 Iba1^+^cells/mm^2 retina [9]). Similarly, in the CCL2-/- and CCR2-/- slow age-related retinal degeneration mouse models, ~ 80–90 Iba1^+^cells/mm^2 were observed in the subretina by the age of 20 months. It is possible that in these slow degenerating models, toxic molecules (e.g., NO, ROS, TNF-α, Il-6, IL-1β [24, 25, 56]) secreted by this relatively small number of subretinal MPs located in close proximity to the photoreceptors, may exacerbate retinal degeneration. This hypothesis should be tested in future studies.

The migration of MPs into the subretina was more pronounced in the central vs. peripheral retina, and minocycline treatment had a more substantial inhibitory effect on the migration of MPs into the peripheral subretina than the central subretina. Several studies reported that the fastest cone loss occurs in the retina’s center in this mouse model [43, 57, 58]. Others have reported that photoreceptor cell debris activates MPs and induces their migration into the subretina [34]. Hence, the faster degeneration of cones in the center of the retina may cause more pronounced MP activation and migration. However, the loss of cones is more rapid in the inferior than the superior hemisphere in the *RPE65/rd12* mouse model [43, 44, 57, 58]. We did not observe a significant difference in MP migration into the subretina between the superior and inferior hemispheres, suggesting that the spatial distribution of MP activation does not entirely match cone photoreceptor degeneration. In the fast retinal degeneration *rd1* mouse model, a central-to-peripheral gradient of MP activation was observed, and it was associated with a central-to-peripheral gradient of photoreceptor loss [28]. A similar central-peripheral gradient in MP migration into the subretina was recently reported in the Lipe −/− mouse model [59]. By contrast, in rats with *rhodopsin*^*P23H*^ mutations, activated MPs are homogeneously distributed throughout the retina, whereas retinal degeneration is more pronounced in the medial compared with the central and peripheral retina [10, 60]. These differences between the spatial association of MP activation and retinal degeneration in the different rodent models suggest that the signals that induce MP activation may differ due to background strain and the mutation-associated disease etiology. The peripheral-to-central gradient of minocycline inhibitory effect on MP migration may have resulted from differences in the bioavailability of minocycline that was administered systemically. Further studies are needed to decipher the molecular mechanism underlying MP activation in the *RPE65/rd12* mouse model.

Our results corroborate similar findings in other mouse models of retinal degeneration. Thus, Du et al. reported that treating *LysMCre-Socs*^*fl/fl*^*Cx3cr1*^*gfp/gfp*^ double knockout mice with minocycline (25 mg/Kg) by gavage for three months resulted in better retinal function (higher ERG a-/b-wave aptitudes) and a thicker retina. Minocycline treatment reduced microglial activation in this mouse model of AMD, lowering the number of microglia cells in the subretina [38]. Similarly, administration of minocycline to *Rho -/-* mice suppressed the migration and accumulation of microglia in the outer retina and elevated photoreceptor survival. However, those authors did not report the spatial distribution of the microglia cells [61]. In addition, they did not characterize the topological distribution of activated microglia cells in the subretina nor the effect of minocycline treatment on microglia cells in the central vs. peripheral retina.

The *RPE65/rd12*,* RPE65-/-*,* LRAT-/-*,* GUCY2D-/-* and the double knock out of *guanylate cyclase-1 (GC1)* and *GC2* genes (*GCdko*) are comparable models of LCA. Cones degenerate quickly in these LCA mouse models, mainly in the peripheral and ventral retina, with almost complete loss of cones by age 4–6 weeks. By contrast, rods have a much slower degeneration rate. Nearly normal ONL thickness is maintained until the age of 7 weeks, and by the age of 6–7 months, the ONL becomes thinner by 30–50% [42, 62–66]. It would be interesting to test whether similar patterns of MP activation and migration are associated with retinal degeneration in the other LCA models. Tang et al. have characterized microglia activation in the CD11c^GFP^Rpe65−/− mouse model. These mice lack RPE65 expression, and a sub-population of microglia cells that possess antigen presentation function expresses high levels of GFP (GFP^hi^). In this mouse model, microglia cells were localized in the inner retina, whereas GFP^hi^ cells were identified in the photoreceptor cell layers at an early age (2 to 4 weeks post-natal) and persisted at the outer retinal layers for at least 14 months. The authors reported that only rare cells were found on the RPE surface, suggesting minimal infiltration of MPs into the subretina in this mouse model. Depletion of microglia and GFP^hi^ cells using Tamoxifen in these mice resulted in a slight increase in cone survival [15]. However, these authors have not presented the effect of Tamoxifen treatment on retinal function. In our study, we used Iba-I staining to identify the MP cells. We found extensive infiltration of MP cells into the subretina and that inhibiting MP activation and subretinal infiltration using minocycline was associated with a significant improvement in retinal function. The differences in MP retinal distribution and therapeutic effects between the study of Tang et al. and our study may have resulted from the differences in the mouse models and the use of different therapeutics targeting MPs.

Our study is limited by the short duration (8 weeks) of minocycline treatment. Most recently, Keenan et al. reported a 24-month minocycline treatment in GA patients. Although no significant improvement in tested outcome measures was observed, these clinical findings support the general safety of minocycline treatment [67]. This is in accordance with our findings that minocycline treatment had no substantial effect on the general health and weight gain of the mice.

Our data suggest that *RPE65/rd12* mice are a good model for studying the therapeutic effect of minocycline and the role of MP activation in retinal degeneration progression. Furthermore, as rod degeneration progresses very slowly in these mice, inhibiting MP activation at different retinal degeneration stages and using various approaches may shed more light on the role of MP activation in retinal degeneration progression.

## Conclusions

Our data suggest that MP activation is associated with diminished retinal function at early stages of retinal degeneration in the *RPE65/rd12* mouse model. Further studies are warranted to evaluate the potential of therapeutics that reduce MP activation, such as minocycline, for retinal neuroprotection.

## Supplementary Information

Below is the link to the electronic supplementary material.

Supplementary Material 1 (PDF 771 KB)

## Data Availability

The datasets generated during and/or analyzed during the current study are available from the corresponding author on reasonable request.
