# Supplementary material for: Minocycline treatment reduces the activation of mononuclear phagocytes and improves retinal function in a mouse model of Leber congenital amaurosis
Source: Graefes Arch Clin Exp Ophthalmol. 2025 Jun 18;263(9):2485–94. doi: 10.1007/s00417-025-06768-y (PMC12513946; doi:10.1007/s00417-025-06768-y)
Supplement: Supplementary file 1 — Supplementary Material 1 (PDF 771 KB) [file 417_2025_6768_MOESM1_ESM.pdf]

**Minocycline treatment reduces the activation of mononuclear phagocytes and improves retinal function in a mouse model of Leber congenital amaurosis.**

Ettel Bubis, Ifat Sher, Hadas Ketter-Katz, Estela Derzane, Florian Sennlaub, Ygal Rotenstreich

# Supplementary Data

**Supplementary Figure 1. Representative dark-adapted ERG waveforms of representative *RPE65/rd12* mice from each study group at P28 and P84.**

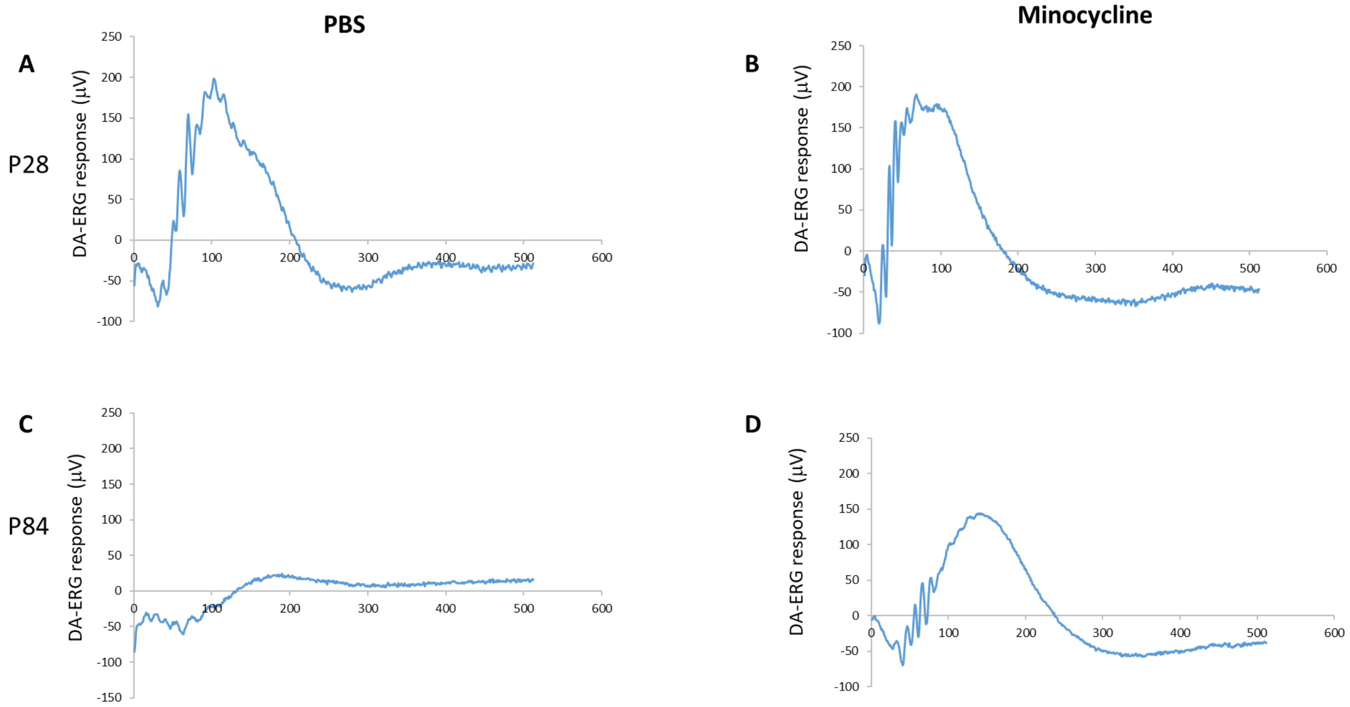


Representative dark-adapted (DA-)ERG waveforms of PBS- (**A, C**) and minocycline (**B, D**)-treated mice at P28 (**A, B**) and P84 (**C, D**).

**Supplementary Figure 2. Representative light-adapted ERG waveforms of representative *RPE65/rd12* mice from each study group at P28 and P84.**

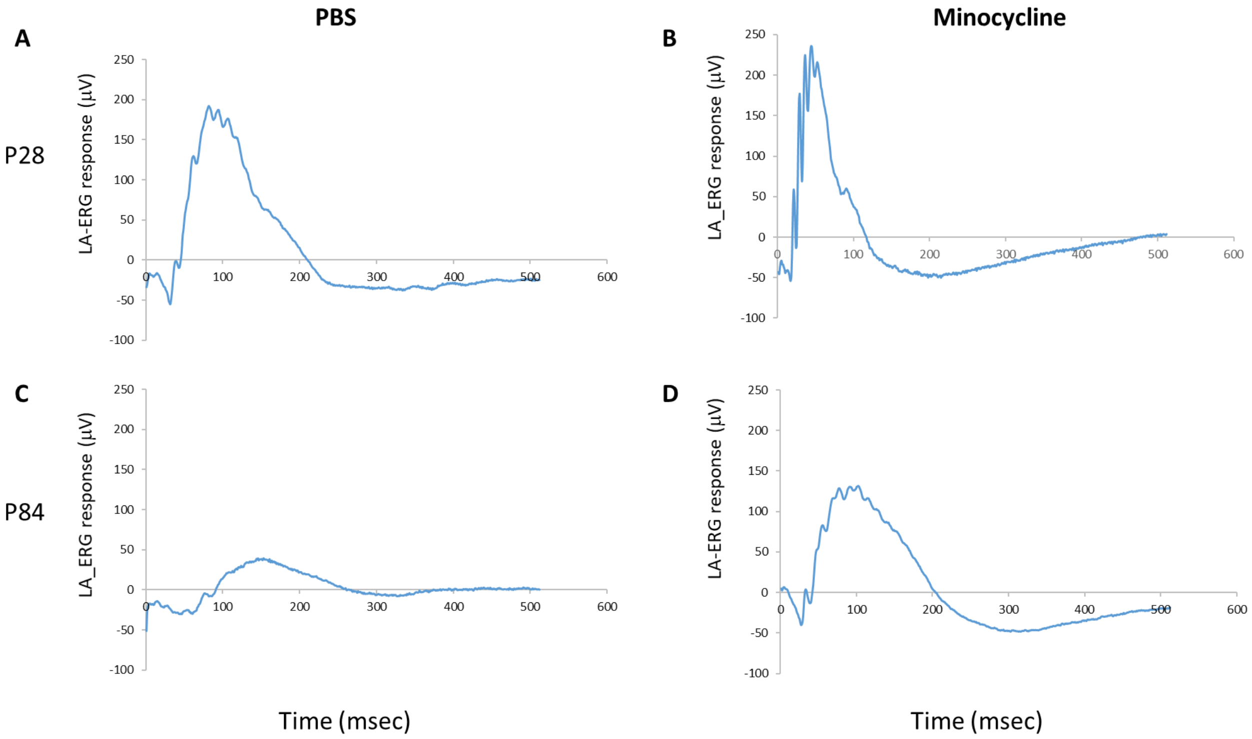


Representative light-adapted (LA)-ERG waveforms of PBS- (**A, C**) and minocycline (**B, D**)-treated mice at  P28 (**A, B**) and P84 (**C, D**).
